# Enhancing the activity of oxygen-evolution and chlorine-evolution electrocatalysts by atomic layer deposition of TiO_2_†

**DOI:** 10.1039/c8ee02351d

**Published:** 2018-12-14

**Authors:** Cody E. Finke, Stefan T. Omelchenko, Justin T. Jasper, Michael F. Lichterman, Carlos G. Read, Nathan S. Lewis, Michael R. Hoffmann

**Affiliations:** a The Linde Center for Global Environmental Science, Caltech, Caltech, Pasadena, CA 91125, USA; b The Resnick Sustainability Institute, Caltech, Caltech, Pasadena, CA 91125, USA; c Division of Engineering and Applied Science, Caltech, Caltech, Pasadena, CA 91125, USA; d Division of Chemistry and Chemical Engineering, Caltech, Caltech, Pasadena, CA 91125, USA

## Abstract

We report that TiO_2_ coatings formed *via* atomic layer deposition (ALD) may tune the activity of IrO_2_, RuO_2_, and FTO for the oxygen-evolution and chlorine-evolution reactions (OER and CER). Electrocatalysts exposed to ~3–30 ALD cycles of TiO_2_ exhibited overpotentials at 10 mA cm^–2^ of geometric current density that were several hundred millivolts lower than uncoated catalysts, with correspondingly higher specific activities. For example, the deposition of TiO_2_ onto IrO_2_ yielded a 9-fold increase in the OER-specific activity in 1.0 M H_2_SO_4_ (0.1 to 0.9 mA cm_ECSA_^–2^ at 350 mV overpotential). The oxidation state of titanium and the potential of zero charge were also a function of the number of ALD cycles, indicating a correlation between oxidation state, potential of zero charge, and activity of the tuned electrocatalysts.

Broader context

Realizing a low anthropogenic CO_2_ emissions future depends on the electrochemical production of fuels and commodity chemicals. In the absence of a substantial carbon tax, electrochemical production of these materials must be cost competitive with conventional production. The levelized cost of electrochemically produced chemicals depends heavily on operational expenses (OpEx; *e.g.*, buying electricity) and the balance of systems costs, and depends relatively less on the price of the catalyst.^1^ Therefore, one pathway to low cost electrochemical fuel and commodity chemical production is to reduce the OpEx by fabricating highly active catalysts. Current methods to enhance catalytic activity are limited or rely on computationally-expensive calculations. Simple tools that can be used to enhance the catalytic activity for a variety of chemical reactions, such as tuning catalysts through atomic layer deposition as presented here, are essential to developing low-cost electrochemical systems that can meet global energy and chemical demands.

## Introduction

Highly active electrocatalysts are required for the cost-effective generation of fuels and commodity chemicals from renewable sources of electricity.^2,3^ Despite potential advantages (*e.g.*, facile product separation), the industrial use of many heterogeneous electrocatalysts is currently limited in part by suboptimal catalytic activity and/or selectivity. In addition, there are limited methods to tune the selectivity and activity of heterogeneous electrocatalysts.^2^ Methods and design tools such as doping, inducing strain, and mixing metal oxides have been used to improve the catalytic activity of heterogeneous electrocatalysts.^4–7^ The activity of heterogeneous electrocatalysts can also be tuned by applying thin layers of another material, leading to an altered surface charge density on the resulting composite material relative to the bulk charge density of either individual material.^8–13^ This approach has been widely used to alter the catalytic and electronic properties of core/shell nanoparticles, although additional tuning of the particle support structure is necessary to create an efficient heterogeneous electrocatalyst.^14,15^ Density functional theory calculations have shown that a single atomic layer of TiO_2_ on RuO_2_ should lead to enhanced selectivity for the chlorine-evolution reaction (CER) relative to the oxygen-evolution reaction (OER).^9^ Enhanced catalytic activity for the OER has been reported for WO_3_ photocatalysts coated with 5 nm of alumina, with the activity increase ascribed to an alteration in the electronic surface-state density.^16^ Enhanced catalytic activity has also been observed at the interface between TiO_2_ and RuO_2_, with charge transfer between RuO_2_ and TiO_2_ resulting in a mixed phasewithanintermediatechargedensity.^5^

Herein, atomic layer deposition (ALD; a stepwise deposition technique) has been used to tune the surface charge density, and consequently tune the catalytic activity, of electrocatalytic systems in a fashion consistent with estimates based on group electronegativity concepts (see Fig. S1–S5 in the ESI† for further discussion of ALD, surface homogeneity, and group electronegativity estimates). To test these predictions, the activities of the known electrocatalysts, IrO_2_, RuO_2_, and F-doped SnO_2_ (FTO) were tuned and evaluated for the chlorine-evolution reaction (CER) and the oxygen-evolution reaction (OER). The CER provides a promising approach to infrastructure-free wastewater treatment as well as for the production of chlorine, an important industrial chemical whose global annual demand exceeds seventy million metric tons.^17,18^ The OER is the limiting half-reaction for water splitting that could provide hydrogen for transportation and could also provide a precursor to energy storage *via* thermochemical reaction with CO_2_ to produce an energy-dense, carbon-neutral fuel.^19^

## Results and discussion

Each material tested was selected based on its theoretical group electronegativity (*χ*) relative to the group electronegativity of RuO_2_ (*χ* ≈ 2.72), the most active catalyst for the OER in the benchmarking literature (Fig. S5, ESI†) as well as the most active catalyst for the CER.^20^ IrO_2_ (*χ* ≈ 2.78) and FTO (*χ* ≈ 2.88) were also investigated because they have higher electronegativities than RuO_2_, and therefore using ALD to overcoat these catalysts with TiO_2_ (*χ* ≈ 2.62) is expected to shift their surface electronic properties (*i.e.*, the potential of zero charge, *E*_ZC_)and catalytic activities towards that of RuO_2_, the optimal single metal oxide catalyst. These materials were also chosen because TiO_2_, IrO_2_, RuO_2_, and other materials are commonly used to form mixed metal oxide electrodes, most notably the dimensionally stable anode (DSA), in which TiO_2_ increases the anode’s stability, but does not confer enhanced activity to the aggregated material.^21^

**Fig. 1:**
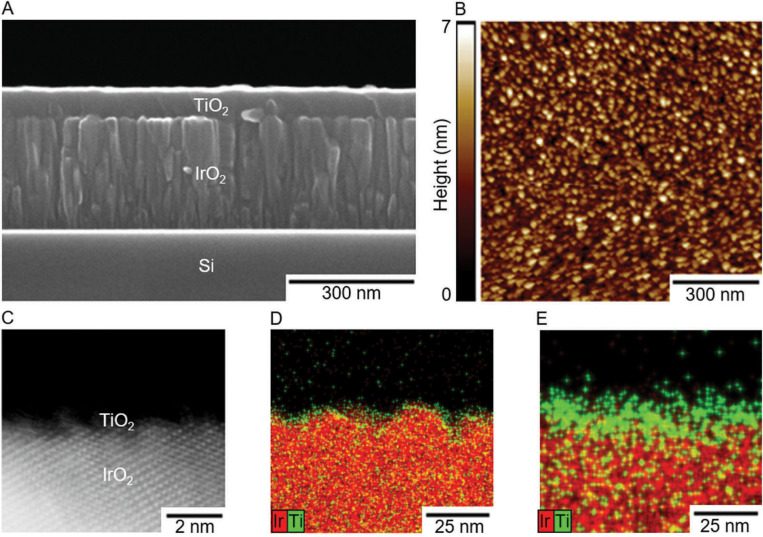
Material characterization of typical electrocatalyst samples. (A) SEM image of an IrO_2_ catalyst with 1000 ALD TiO_2_ cycles. (B) AFM map of IrO_2_ with 10 ALD cycles of TiO_2_. (C) HAADF-STEM image of an IrO_2_-based electrocatalyst with 10 ALD cycles of TiO_2_. The underlying crystalline material is IrO_2_ while the hair-like material at the surface is TiO_2_. (D and E) Energy dispersive X-ray spectroscopy (EDS) maps of IrO_2_-based electrocatalysts with 10 and 40 ALD cycles of TiO_2_, respectively. The red color indicates Ir and green indicates Ti. Note that green and red intermix throughout this cross section due to the inherent roughness of the sample.

Overpotentials (*η*; the excess potential beyond the equilibrium potential required to reach a given current density) were determined for IrO_2_, RuO_2_, and FTO as a function of the successive number of TiO_2_ ALD cycles (see ESI† for additional details on electrode preparation and testing, and TiO_2_ growth rate) for the OER at 10 mA (cm_geo_)^–2^ in 1.0 M H_2_SO_4_ and for the CER at 1 mA (cm_geo_)^–2^ in 5.0 M NaCl adjusted to pH 2.0 with HCl. Current densities were chosen to produce >95% measured Faradaic efficiency for each catalyst (Table S2, ESI†), and current–potential data were corrected for the solution resistance (<2.0 mV correction) as measured by electrochemical impedance spectroscopy (see ESI† for details). The three catalysts were prepared on substrates that had very low roughness to minimize effects in geometric overpotential measurements due to surface area differences. Specifically, electrocatalyst samples consisted of a ~300 nm metal–oxide film sputter deposited on a (100)-oriented Si substrate, in the case of IrO_2_ and RuO_2_, or commercially available TEC 15 FTO glass substrates, in the case of FTO-based electrocatalysts. TiO_2_ overlayers were then deposited on top of the electrocatalysts. The microstructure of a typical IrO_2_-based electrocatalyst is shown in the cross-sectional scanning electron microscopy (SEM) image in Fig. 1A. The resulting electrocatalysts were very smooth with low surface roughness (Fig. 1B) such that the surface area as measured by atomic-force microscopy (AFM) was roughly equivalent to the measured geometric surface areas (Table S1, ESI†). Further characterization of the electrocatalysts’ surface topology can be found in Fig. S1–S4 and Table S1 (ESI†).

**Fig. 2:**
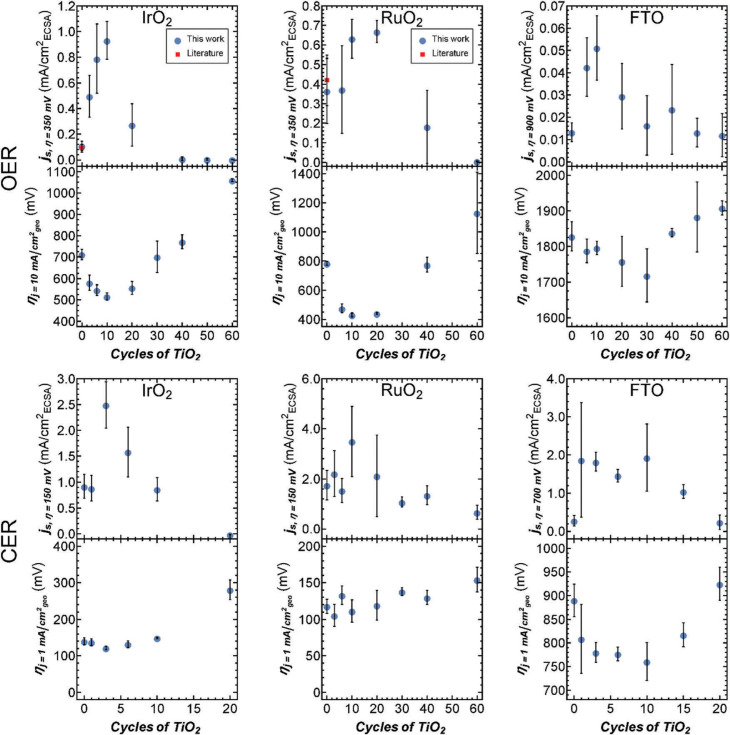
Specific activities (*j_s_*) and overpotentials (*η*) for the OER and CER on IrO_2_, RuO_2_, and FTO coated at various ALD cycles of TiO_2_. Overpotentials were measured at 10 mA (cm_geo_)^–2^ for the OER and at 1 mA (cm_geo_)^–2^ for the CER (normalized to geometric surface area). Specific activities for the OER were measured at 350 mV (IrO_2_ and RuO_2_) or 900 mV (FTO). Specific activities for the CER were measured at 150 mV (IrO_2_ and RuO_2_) or 700 mV (FTO). The red squares indicate available literature values.

Geometric overpotentials for these catalysts were considerably higher than geometric overpotentials for identical catalysts prepared on rougher substrates, however, the measured OER overpotentials at 10 mA (cm_geo_)^–2^ for bare RuO_2_ and IrO_2_ agreed well with values reported for catalysts prepared on similarly flat surfaces. We are unaware of comparable OER data for FTO or for CER catalysts.^20,22^ The overpotentials for IrO_2_ and FTO, for both the OER and CER, initially showed an improvement (*i.e.*, reduction) with increasing ALD cycle number, before exhibiting an inflection point due to an increase in overpotential at higher ALD cycle numbers (Fig. 2). The triangular shape observed between the overpotential and the TiO_2_ ALD cycle number is typical of a volcano-type relationship that exemplifies the Sabatier principle.^23^ The overpotential reductions between bare IrO_2_ and FTO catalysts and those at the peak of the volcano curve for the OER were Δ*η*_OER_ ≈ –200 mV at 10 cycles and –100 mV at 30 cycles, respectively. For the CER, the observed overpotential reductions were Δ*η*_CER_ ≈ –30 mV at 3 cycles and –100 mV at 10 cycles, for IrO_2_ and FTO respectively (Fig. 2). A volcano-type relationship between cycle number and overpotential was also observed for RuO_2_ facilitating the OER, with Δ*η*_OER_ ≈ –350 mV between 0 and 10 cycles. However, for the CER, the overpotential of the RuO_2_-based catalyst increased with TiO_2_ ALD cycle number (Fig. 2).

The specific activity (*i.e.*, the current density normalized to the electrochemically active surface area (ECSA)) is a standard quantity for comparing the OER activity of heterogeneous electrocatalysts (see Fig. S9–S11, and the ESI† for details on specific activity calculations and additional discussion). For IrO_2_ and RuO_2_ catalysts, the OER specific activities of the uncoated catalysts were in good agreement with previously reported values.^20^ We are unaware of reported specific activities for FTO for the OER or for any catalyst for the CER. The specific activities for the OER and CER were characterized by volcano-type relationships as a function of the TiO_2_ ALD cycle number (Fig. 2). In fact, IrO_2_ coated with 10 ALD cycles of TiO_2_ showed a 9-fold increase in OER specific activity at *η* = 350 mV relative to uncoated IrO_2_. Recently, IrO_*x*_/SrIrO_3_ has been reported as an especially active catalyst using current normalized to atomic force microscopy measured surface area (AFMSA) in 0.5 M H_2_SO_4_. To compare these catalysts, we measured the roughness of our catalysts using AFM (Table S1, ESI†). For our catalysts, bare IrO_2_ exhibited a Tafel slope of ~60 mV dec^–1^ in good agreement with previously reported OER catalysts.^24^ As the activity of our IrO_2_ based catalyst increased from bare IrO_2_ to 10 TiO_2_ ALD cycles, the Tafel slope remained constant at ~60 mV dec^–1^ while the exchange current density (*i*_0_)increased from ~1 × 10^–7^ to ~2 × 10^–5^ mA (cm_AFMSA_)^–2^. Initially the IrO_*x*_/SrIrO_3_ catalyst also had an OER Tafel slope of ~60 mV dec^–1^ and an *i*_0_ of ~7 × 10^–6^ mA (cm_AFMSA_)^–2^. For the IrO_*x*_/SrIrO_3_, however, after a period of activation the Tafel slope improved dramatically to ~40 mV dec^–1^, which indicates a previously unknown OER mechanism, while the *i*_0_ deteriorated to ~3 × 10^–7^ mA (cm_AFMSA_)^–2^ (see Fig. S11, Table S5, and ESI† for details on Tafel analysis). In our case, IrO_2_ coated with 10 ALD cycles of TiO_2_ exhibited lower overpotentials than the freshly prepared IrO_*x*_/SrIrO_3_ catalyst at current densities <1 mA (cm_AFMSA_)^–2^ and lower overpotentials than the activated IrO_*x*_/SrIrO_3_ catalyst at <0.02 mA (cm_AFMSA_)^–2^, but substantially higher overpotentials at the more industrially relevant current densities of >10 mA (cm_AFMSA_)^–2^.^2,25^ Further discussion on surface roughness, including AFM, and SEM sample characterization is presented in the ESI† (Fig. S1–S4 and Table S1).

To test the longevity of the enhanced catalytic performance with TiO_2_ deposition, we performed 24 h stability testing at 10 mA cm^–2^ for both the CER and the OER for the uncoated catalyst and for the most active catalyst for each material system. The catalysts investigated herein were not optimized for stability and, as was previously reported for thin IrO_2_ and RuO_2_ catalyst depositions,^20,26^ the overpotential on uncoated catalysts for the OER in 1 M H_2_SO_4_ degraded rapidly after <1h of operation at 10 mA (cm_geo_)^–2^. For thinly coated catalysts (3–10 cycles) the OER stability improved from about 1 h to about 4 h, while for thicker TiO_2_ coatings (>30 cycles) the OER stability increased to >9 h (Fig. S7, ESI†). The loss in activity for the OER for TiO_2_ coated samples was associated with a loss in the TiO_2_ coating as illustrated in X-ray photoelectron spectroscopy (XPS) measurements of the Ti 2p core level before and after electrochemical stability testing (Fig. S22, ESI†). For the CER, all catalysts were relatively stable over the 24 h testing period except for the FTO-based catalysts which followed the same trend as the OER, with thicker TiO_2_ coatings stabilizing the electrodes. XPS measurements of the stable CER catalysts indicated that the TiO_2_ overcoating was still present even after 24 h of continuous operation (Fig. S23, ESI†). These results indicate that, as prepared here, these catalysts are not long-term stable, and substantial work is needed to obtain an industrially relevant catalyst. Similarly prepared catalysts exhibit enhanced stability by making the catalyst material thicker, annealing the catalyst, or mixing Sb_*x*_O_*y*_, TiO_2_, Ta_*x*_O_*y*_, or SnO_2_ into the catalyst.^26–28^ It is possible that similar techniques could be used to enhance the stability of the catalysts presented in this work.

The enhancement in catalytic performance observed with deposition of TiO_2_ is not readily explained by surface morphological changes of the electrocatalyst. Deposition of TiO_2_ does not substantially affect the electrochemically active surface area, a metric believed to be related to active site density, and changes in the surface area alone do not account for the magnitude of the enhancement in the specific activity (Fig. S11, ESI†). Furthermore, while high-angle annular dark-field scanning transmission electron microscopy (HAADF-STEM) images and STEM electron dispersive X-ray spectroscopy (EDS) maps of IrO_2_ samples with 10 cycles of TiO_2_ (Fig. 1C and D) indicate that the TiO_2_ film is semi-continuous with small areas of the underlying IrO_2_ exposed, deposition of 40 cycles of TiO_2_ results in a uniform, continuous film (Fig. 1E) and catalysis commensurate with the bare IrO_2_ samples. These facts suggest the phenomenon does not arise from surface morphological effects alone, instead suggesting that TiO_2_ is playing a partial role in enhancing the activity of the active sites. The idea that TiO_2_ may be able to play a role in the active site is consistent with both experimental and computational literature which indicates that TiO_2_ may hydrate and evolve both chlorine and oxygen.^3,29–31^ The Tafel slopes for all active IrO_2_ and RuO_2_ based catalysts agree well with previously reported Tafel slopes (~60 mV dec^–1^ and ~30 mV dec^–1^ for the OER and CER respectively; Tables S5, S6 and Fig. S11, ESI†),^32^ consistent with expectations that addition of TiO_2_ does not fundamentally change the mechanism or the potential determining step for either reaction. Hypothesized mechanisms generally involve coordination of either OOH or OCl groups to unsaturated sites on the metal oxide in the potential determining reaction steps.^33–35^ FTO based catalysts exhibited very large overpotentials for both the CER and OER and had correspondingly high Tafel slopes in excess of 190 mV dec^–1^, potentially indicating a different, much less efficient mechanism than the process that controls the reactivity of the more active catalysts.

**Fig. 3:**
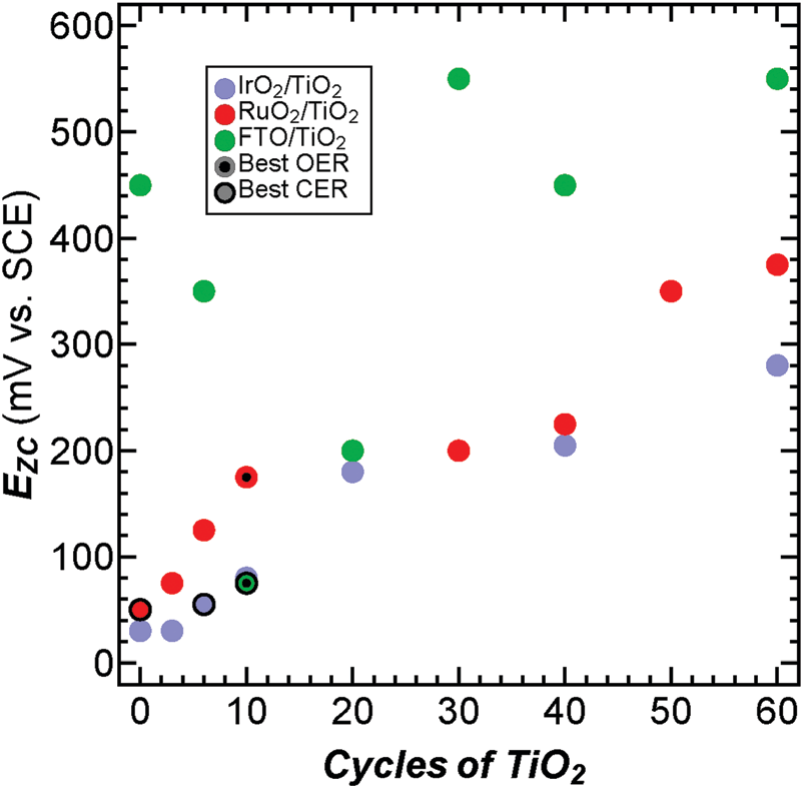
*E*_ZC_ of IrO_2_ (blue), RuO_2_ (red), and FTO (green) anodes coated with various ALD cycles of TiO_2_. Black dots and circles with black borders indicate the catalysts with the highest specific activity for each catalyst for the OER and CER, respectively.

To investigate the electrocatalysts’ surface electronic properties the potentials of zero charge (*E*_ZC_) of the electrocatalysts were measured as a function of TiO_2_ thickness (Fig. 3). *E*_ZC_ is the potential that must be applied to produce a neutral surface and is an indicator of a material’s willingness to lose electrons, with more positive *E*_ZC_ values indicating surfaces that are less willing to lose their electrons (see ESI,† eqn (S2) and (S3) and Fig. S12–S15 for details and discussion on handling thin TiO_2_ layers in *E*_ZC_ measurements). *E*_ZC_ thus yields insight into the strength of the bonds on the catalyst surface.^36,37^ *E*_ZC_ is also qualitatively very similar to group electronegativity which describes how difficult it is for molecules to gain electrons and is correlated to OER activity (Fig. S5, ESI†). Additionally, *E*_ZC_ of metal electrodes has been correlated with metal–oxygen single bond strengths which is also qualitatively similar to computationally derived oxygen binding energies which have long been correlated with electrocatalytic activity.^2,36,38^ *E*_ZC_, group electronegativity, and oxygen binding energies each have their strengths and weaknesses. *E*_ZC_ is measurable, but it is not easy to predict. Electronegativity is completely theoretical and very simple to calculate, but does not take into account more complex qualities of materials like edge sites. Oxygen binding energies are strongly theoretically grounded and can take into account complexities of materials like edge sites, but they are also relatively difficult to calculate. These strengths and weaknesses show that all these descriptors may be used complimentarily to predict and understand catalytic activity (Fig. S5, ESI†). Measured *E*_ZC_ values for bare RuO_2_ and IrO_2_ (50 and 30 mV *vs*. SCE, respectively) were consistent with previously reported values for Ru and Ir.^39^ We are unaware of reported *E*_ZC_ values for FTO. As the RuO_2_ and IrO_2_ samples were coated with increasing ALD cycles of TiO_2_ the *E*_ZC_ shifted from lower to higher potentials in both cases and eventually reached the value for bulk TiO_2_. This behavior is consistent with the expected trends for equilibrated group electronegativities. The *E*_ZC_ for bare FTO (450 mV *vs*. SCE) was less than that for bulk TiO_2_ and greater than bare IrO_2_ or RuO_2_. The FTO *E*_ZC_ decreased with increasing TiO_2_ cycles up to 10 cycles and as the TiO_2_ cycles increased beyond 10 the *E*_ZC_ increased until it reached the bulk value of TiO_2_ at large cycle numbers. The overall trend of the FTO *E*_ZC_ increasing to higher values with increasing TiO_2_ cycle number is consistent with group electronegativity arguments. However, the intermediate behavior where the *E*_ZC_ decreases and then increases is not well explained by group electronegativity and could, in part, arise from the complicated behavior of the F dopant atoms (further discussion on the limits of group electronegativity are found in the ESI†). For all catalysts, the *E*_ZC_ continued to shift even beyond the point where TEM data indicated that the film is continuous (40 ALD cycles). This suggests that the exposed metal oxide is not fully responsible for the shift in *E*_ZC_ and that the surface TiO_2_ is likely responsible in part for the *E*_ZC_ shift. Shifts in *E*_ZC_ with incremental TiO_2_ deposition suggest that ALD can be used to tune the catalytic performance. These data reveal that the catalysts with the highest activity for the CER have *E*_ZC_ values between 50 and 75 mV *vs*. SCE (Fig. 3), consistent with the observation that addition of TiO_2_ layers to RuO_2_ decreased the activity of RuO_2_ electrocatalysts (*E*_ZC_ =50 mV *vs*. SCE) for the CER. Additionally, active OER and CER catalysts for all systems investigated have *E*_ZC_ values between 25 and 200 mV *vs*. SCE with the best OER catalysts having a somewhat higher *E*_ZC_ (~110 mV *vs*. SCE) than the best CER catalysts (~60 mV *vs*. SCE).

**Fig. 4:**
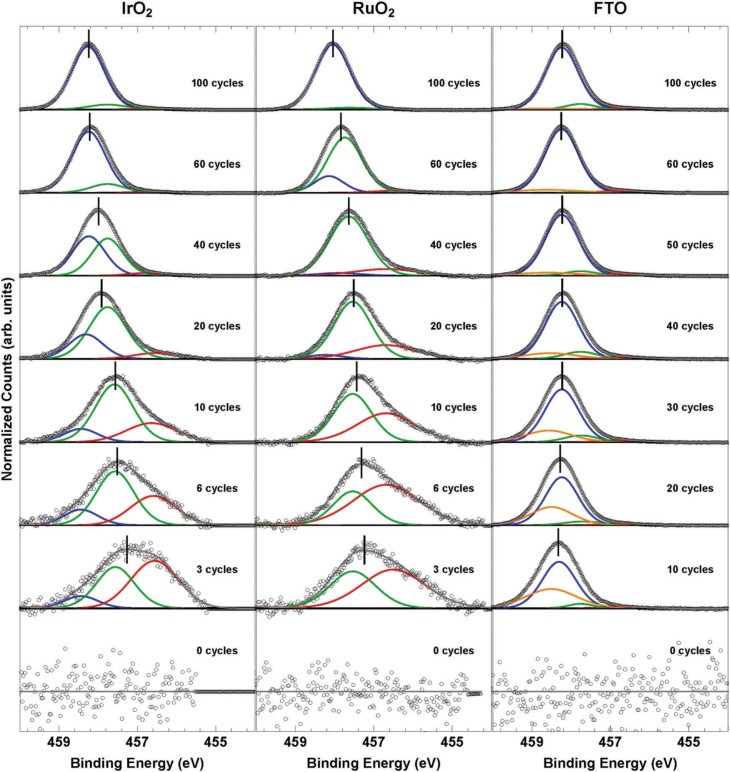
X-ray photoelectron spectroscopy of the Ti 2p_3/2_ region for IrO_2_, RuO_2_, and FTO catalysts with varying TiO_2_ thicknesses. Bulk TiO_2_ is shown as the blue peak in each spectrum. The slightly and highly reduced Ti peaks are shown in green and red, respectively, and the most highly oxidized Ti peak is shown in orange.

To further understand the surface states of the catalysts, X-ray photoelectron spectroscopy was used to measure the Ti oxidation state. Fig. 4 shows the Ti 2p_3/2_ core-level photoemission (for the full Ti 2p region see Fig. S16, ESI†), stacked from bottom to top, for increasing ALD TiO_2_ thickness, with 0 cycles indicating the bare catalyst substrate. Deposition of low cycle numbers of ALD TiO_2_ on IrO_2_ and RuO_2_ produced Ti core-level peaks that were at ~456.6 eV and ~457.6 eV, which is consistent with previously reported binding energies for Ti^3+^ states.^40,41^ As the ALD cycle number increased, the Ti oxidation state for these samples gradually increased to its bulk oxidation state (~+4), and signals indicative of bulk TiO_2_ were eventually observed (Fig. 4). In the case of ALD TiO_2_ on FTO, the lower cycle number thicknesses instead produced binding energies primarily at the bulk position, in addition to a peak at a higher binding energy. This additional peak can be ascribed to a mixed phase between the substrate (FTO) and the thin TiO_2_ film, in which the chemical nature of the phase produces a more oxidized metal, with the mixed phase most likely dominated by Ti^4+^ sites.

The variation in the Ti oxidation state with ALD TiO_2_ cycles was accompanied by a peak shift of the Ti 2p_3/2_ peak relative to the bulk TiO_2_ peak position (Fig. S19, ESI†). The Ti 2p_3/2_ peak of the IrO_2_-and RuO_2_-based catalysts shifted from reduced, lower binding energies to the more oxidized, higher binding energies typical of bulk TiO_2_. The FTO-based Ti 2p_3/2_ peak shifts from more oxidized, high binding energies at low TiO_2_ cycles to lower binding energies for intermediate TiO_2_ cycles (10–40 cycles) before increasing again to higher binding energies at large TiO_2_ thicknesses (>60 cycles). The Ti 2p_3/2_ peak shift is qualitatively consistent with the variation in *E*_ZC_ with TiO_2_ cycle number suggesting that the change in the surface charge density is correlated with a change in the Ti oxidation state.

The variation in the Ti oxidation state with TiO_2_ thickness can be explained by charge transfer from the underlying metal oxide substrate. In this scenario, a more reduced Ti species present at low deposited cycles of TiO_2_ on IrO_2_ and RuO_2_ would be accompanied by a more oxidized metal oxide substrate. To confirm this hypothesis, we measured the Ir 4f, Ru 3d, and Sn 3d core-level photoemission (Fig. S20, ESI†). Unlike in the case of the Ti 2p spectra, the Ir 4f, Ru 3d, and Sn 3d core-level photoemission exhibited very small changes between the bare metal oxide substrate and those with varying thicknesses of TiO_2_. This was reflected in the peak shifts of the main peak for the Ir 4f, Ru 3d, and Sn 3d spectra with TiO_2_ thickness relative to that of the bare substrate (Fig. S21, ESI†), which were an order of magnitude lower than those for the Ti 2p core-level photoemission and mostly within the error of the measurement (±0.1 eV). While peak fitting (see the ESI† for details) of these spectra indicates that initial deposition of TiO_2_ leads to a slightly more oxidized Ir and Ru state, and a slightly more reduced Sn state for FTO, no trend with thickness was observed for any of the substrates, and changes in the oxidation state of the underlying catalyst are likely below the detection limit for the techniques used in this study (Fig. S20, S21 and Table S7, ESI†).

## Conclusion

In summation, surface characterization suggests that atomic layer deposition of low cycle numbers of TiO_2_ can tune surface electron densities of the catalyst in a direction consistent with predictions from group electronegativity concepts (Fig. S5, ESI†). Given that concomitant changes in electrochemical activity were observed with deposition of TiO_2_, these data indicate that ALD may be useful to tune the activity of other catalysts for diverse reactions, including those critical for renewable energy storage and wastewater treatment.

## Notes and references

1 M. R. Shaner, H. A. Atwater, N. S. Lewis and E. W. McFarland, *Energy Environ. Sci.*, 2016, **9**, 2354–2371.

2 Z. W. Seh, J. Kibsgaard, C. F. Dickens, I. Chorkendorff, J. K. Nørskov and T. F. Jaramillo, *Science*, 2017, **355**, eaad4998.

3 Y. Yang, J. Shin, J. T. Jasper and M. R. Hoffmann, *Environ. Sci. Technol.*, 2016, **50**, 8780–8787.

4 K. Gong, F. Du, Z. Xia, M. Durstock and L. Dai, *Science*, 2009, **323**, 760–764.

5 L.-Å. Näslund, C. M. Sánchez-Sánchez, Á. S. Ingason, J. Bäckström, E. Herrero, J. Rosen and S. Holmin, *J. Phys. Chem. C*, 2013, **117**, 6126–6135.

7 H. Li, C. Tsai, A. L. Koh, L. L. Cai, A. W. Contryman, A. H. Fragapane, J. H. Zhao, H. S. Han, H. C. Manoharan, F. Abild-Pedersen, J. K. Norskov and X. L. Zheng, *Nat. Mater.*, 2016, **15**, 48–53.

8 L. Giordano, F. Cinquini and G. Pacchioni, *Phys. Rev. B: Condens. Matter Mater. Phys.*, 2006, **73**, 045414.

9 K. S. Exner, J. Anton, T. Jacob and H. Over, *Angew. Chem., Int. Ed.*, 2014, **53**, 11032–11035.

13 R. T. Sanderson, *Chemical periodicity*, Reinhold Pub. Corp., New York, 1960.

14 Z. Zhuang, W. Sheng and Y. Yan, *Adv. Mater.*, 2014, **26**, 3950–3955.

15 L. Bu, N. Zhang, S. Guo, X. Zhang, J. Li, J. Yao, T. Wu, G. Lu, J.-Y. Ma, D. Su and X. Huang, *Science*, 2016, **354**, 1410–1414.

16 W. Kim, T. Tachikawa, D. Monllor-Satoca, H.-i. Kim, T. Majima and W. Choi, *Energy Environ. Sci.*, 2013, **6**, 3732–3739.

17 K. Cho, D. Kwon and M. R. Hoffmann, *RSC Adv.*, 2014, **4**, 4596–4608.

18 Y. P. Khalil, *Engineering*, 2015, **360**.

19 M. A. Pellow, C. J. M. Emmott, C. J. Barnhart and S. M. Benson, *Energy Environ. Sci.*, 2015, **8**, 1938–1952.

20 C. C. L. McCrory, S. Jung, I. M. Ferrer, S. M. Chatman, J. C. Peters and T. F. Jaramillo, *J. Am. Chem. Soc.*, 2015, **137**, 4347–4357.

21 S. Trasatti, *Electrochim. Acta*, 2000, **45**, 2377–2385.

22 K. A. Stoerzinger, L. Qiao, M. D. Biegalski and Y. Shao-Horn, *J. Phys. Lett.*, 2014, **5**, 1636–1641.

23 A. J. Medford, A. Vojvodic, J. S. Hummelshøj, J. Voss, F. Abild-Pedersen, F. Studt, T. Bligaard, A. Nilsson and J. K. Nørskov, *J. Catal.*, 2015, **328**, 36–42.

24 J.-M. Hu, J.-Q. Zhang and C.-N. Cao, *Int. J. Hydrogen Energy*, 2004, **29**, 791–797.

25 L. C. Seitz, C. F. Dickens, K. Nishio, Y. Hikita, J. Montoya, A. Doyle, C. Kirk, A. Vojvodic, H. Y. Hwang, J. K. Norskov and T. F. Jaramillo, *Science*, 2016, **353**, 1011–1014.

26 C. C. L. McCrory, S. Jung, J. C. Peters and T. F. Jaramillo, *J. Am. Chem. Soc.*, 2013, **135**, 16977–16987.

28 G. P. Vercesi, J. Rolewicz, C. Comninellis and J. Hinder, *Thermochim. Acta*, 1991, **176**, 31–47.

29 K. Cho and M. R. Hoffmann, *Chem. Mater.*, 2015, **27**, 2224–2233.

31 Y. Yang and M. R. Hoffmann, *Environ. Sci. Technol.*, 2016, **50**, 11888–11894.

32 S. Trasatti, *Electrochim. Acta*, 1984, **29**, 1503–1512.

33 M. García-Mota, A. Vojvodic, H. Metiu, I. C. Man, H.-Y. Su, J. Rossmeisl and J. K. Nørskov, *ChemCatChem*, 2011, **3**, 1607–1611.

35 I. C. Man, H.-Y. Su, F. Calle-Vallejo, H. A. Hansen, J. I. Martínez, N. G. Inoglu, J. Kitchin, T. F. Jaramillo, J. K. Nørskov and J. Rossmeisl, *ChemCatChem*, 2011, **3**, 1159–1165.

36 S. Trasatti, *J. Electroanal. Chem. Interfacial Electrochem.*, 1971, **33**, 351–378.

37 S. Trasatti, *J. Electroanal. Chem. Interfacial Electrochem.*, 1972, **39**, 163–184.

38 V. Dimitrov and T. Komatsu, *J. Solid State Chem.*, 2012, **196**, 574–578.

39 A. N. Frumkin and O. A. Petrii, *Electrochim. Acta*, 1975, **20**, 347–359.

40 F. Werfel and O. Brummer, *Phys. Scr.*, 1983, **28**, 92–96.

41 D. Gonbeau, C. Guimon, G. Pfisterguillouzo, A. Levasseur, G. Meunier and R. Dormoy, *Surf. Sci.*, 1991, **254**, 81–89.

